# Network analysis of drug effect on triglyceride-associated DNA methylation

**DOI:** 10.1186/s12919-018-0130-0

**Published:** 2018-09-17

**Authors:** Elise Lim, Hanfei Xu, Peitao Wu, Daniel Posner, Jiayi Wu, Gina M. Peloso, Achilleas N. Pitsillides, Anita L. DeStefano, L. Adrienne Cupples, Ching-Ti Liu

**Affiliations:** 1 Department of Biostatistics, Boston University, 801 Massachusetts Avenue 3rd Floor, Boston, MA 02118 USA; 2 Department of Genetics and Genomics, Boston University, 72 East Concord Street, Boston, MA 02118 USA

## Abstract

**Background:**

DNA methylation, an epigenetic modification, can be affected by environmental factors and thus regulate gene expression levels that can lead to alterations of certain phenotypes. Network analysis has been used successfully to discover gene sets that are expressed differently across multiple disease states and suggest possible pathways of disease progression. We applied this framework to compare DNA methylation levels before and after lipid-lowering medication and to identify modules that differ topologically between the two time points, revealing the association between lipid medication and these triglyceride-related methylation sites.

**Methods:**

We performed quality control using beta-mixture quantile normalization on 463,995 cytosine-phosphate-guanine (CpG) sites and deleted problematic sites, resulting in 423,004 probes. We identified 14,850 probes that were nominally associated with triglycerides prior to treatment and performed weighted gene correlation network analysis (WGCNA) to construct pre- and posttreatment methylation networks of these probes. We then applied both WGCNA module preservation and generalized Hamming distance (GHD) to identify modules with topological differences between the pre- and posttreatment. For modules with structural changes between 2 time points, we performed pathway-enrichment analysis to gain further insight into the biological function of the genes from these modules.

**Results:**

Six triglyceride-associated modules were identified using pretreatment methylation probes. The same 3 modules were not preserved in posttreatment data using both the module-preservation and the GHD methods. Top-enriched pathways for the 3 differentially methylated modules are sphingolipid signaling pathway, proteoglycans in cancer, and metabolic pathways (*p* values < 0.005). One module in particular included an enrichment of lipid-related pathways among the top results.

**Conclusions:**

The same 3 modules, which were differentially methylated between pre- and posttreatment, were identified using both WGCNA module-preservation and GHD methods. Pathway analysis revealed that triglyceride-associated modules contain groups of genes that are involved in lipid signaling and metabolism. These 3 modules may provide insight into the effect of fenofibrate on changes in triglyceride levels and these methylation sites.

## Background

Epigenetic changes are biochemical modifications in chromosomes that do not alter the DNA sequence [1]. DNA methylation is one such epigenetic process implicated in human disease. DNA methylation involves the addition of a methyl group to DNA, which usually occurs at cytosine-phosphate-guanine (CpG) dinucleotides in the promoter region or within genes [1]. It is known to regulate gene expression levels by changing the chromatin structure, thereby preventing transcription factors from binding to the gene promoter, which can lead to alterations of phenotypes [2]. DNA methylation can be modulated by external factors, such as smoking or exposure to toxins [3]. As such, epigenetic information is considered to be fundamental in understanding the interaction between the human genome and the environment.

Recent research has unveiled the potential involvement of DNA methylation on the regulation of fasting blood lipids [4–6]. One way to visualize interactions and changes in the DNA methylation profile is to construct methylation networks and compare their topology. Structural changes resulting from external stimuli can be detected with network comparison algorithms that can identify subnetworks that are either preserved or structurally different. Several papers have performed network-based methods to identify trait-related modules [7–9] and examined the preservation of such modules, either between different tissues or different data sets. For example, Horvath et al. [7] conducted weighted gene correlation network analysis (WGCNA) to examine the effect of aging on DNA methylation modules in humans and reported a robustly defined age-related comethylation module that is present in multiple human issues including blood and brain. Rickabaugh et al. [8] also found a preserved methylation module that was associated with age and HIV-1 status in 2 different HIV data sets via WGCNA. However, to our best knowledge, there are no previous studies using network-based approaches to identify triglyceride-associated modules and assess topological differences for each module between 2 time points. We investigated the topological differences of triglyceride-associated methylation networks constructed before and after the administration of fenofibrate.

## Methods

### Quality control

Data from GAW20 was used and included epigenetic and pharmacogenomics data for 188 unique families [10]. A total of 995 individuals with pretreatment DNA methylation profiles at visit 2 and 530 individuals with posttreatment DNA methylation profiles at visit 4 (with 446 individuals overlapping during the 3-week treatment period) were used to construct, separately, a pre- and posttreatment network. Because of systemic differences in the range of Infinium I and Infinium II probe methylation values, we performed beta-mixture quantile normalization (BMIQ) using the *bmiq* function from *wateRmelon* package in R on a total of 463,995 methylation probes to adjust the beta values of Type II probes to align with the distribution of Type I probes [11, 12]. Probes with single nucleotide polymorphisms (SNPs) under the actual CpG sites, SNPs at the nucleotide right next to a CpG site, or cross-reactive probes that have a target sequence similar to another location in the genome are potentially problematic, and were excluded from analysis [13]. A total of 423,004 pretreatment CpG sites were used in the analysis.

### Network

We were interested in DNA methylation changes caused by fenofibrate. To build the networks, we limited the methylation probes to those that are nominally associated (*p* < 0.05) with the log-transformed triglyceride level from visit 2, resulting in 14,850 probes from pretreatment data. If we limited our study to those probes that are significantly associated (false discovery rate [FDR] < 0.05), we might lose many informative probes that only show nominal significance without considering their potential interactions with other probes used in the network constructions.

To isolate the treatment effect from other covariates, we built the networks from pre- and posttreatment residual methylation values. We performed linear mixed models using the *lmekin* function in the *coxme* package in R for each of the 14,850 pretreatment CpG sites, adjusting for family relatedness, the first 10 principal components accounting for residual batch effects, and the covariates age, sex, field center, and smoking status [14]. Similarly, we obtained residuals for posttreatment methylation for the same set of CpG sites. Samples with more than half missing residuals and CpG sites with variance smaller than 1 × 10^− 10^*max *(abs [residuals of all probes among all samples])* were removed. We applied hierarchical clustering to samples to detect sample outliers at a branch cut height of 7 using the *hclust* function from the *stats* package in R. Overall, 47 probes and 3 samples were excluded in both pre- and posttreatment methylation data.

We used the residuals to construct pretreatment and posttreatment networks using WGCNA, which uses pairwise correlations between the variables to create a weighted network [15]. We chose soft thresholding power β according to the approximate scale-free topology criterion and computed the adjacency matrix by raising co-expression similarities by power β [16]. We chose a power of β = 3 as it was the lowest power at which the scale-free topology had a good fit of R^2^ greater than 0.9.

To minimize the effects of noise and spurious associations, the adjacency matrix was transformed into the topological overlap matrix, which reflects the relative interconnectedness between a pair of methylation probes, and the corresponding dissimilarity matrix was calculated by subtracting the adjacency matrix from 1. We performed hierarchical clustering of the dissimilarity matrix and a dynamic tree-cut algorithm to identify modules in the pretreatment network, with a minimum module size of 30 to avoid small modules and pass more informative results to the subsequent pathway analysis [17, 18]. Transformation of the adjacency matrix and network construction were performed using the *blockwiseModules* function from *WGCNA* package in R [15].

We tested whether pretreatment methylation modules changed after treatment more than we would expect by chance using the module-preservation method and the generalized Hamming distance (GHD) method. The module-preservation statistic is comprised of many factors that describe the density of the network and network connectivity. These statistics are aggregated and were used to calculate the z-score, *Z*_*summary,*_ for each preservation measure [19]. A *Z*_*summary*_ > 10 indicated strong evidence of module preservation, whereas a *Z*_*summary*_ between 2 and 10 indicated weak evidence of preservation. If *Z*_*summary*_ was < 2, we concluded there was no evidence of module preservation. Module changes were also ranked according to a rank statistic *medianRank*, which is also composed of statistics related to a network’s density and connectivity, but is less sensitive to module size according to Langfelder et al. [19]. We used both statistics to determine whether or not a certain module was preserved between pre- and posttreatment. Module preservation analyses were conducted using the *modulePreservation* function from the *WGCNA* package in R [15]*.* GHD is a distance metric that uses one-step topological overlap as the edge weights to account for its direct neighboring structure around the pair of nodes. Formal hypothesis testing can be done by finding the null distribution using permutation, but it is computationally inefficient. In a scale-free network, GHD permutation distribution is approximately normally distributed, ie, ${GHD}_{\pi}\left({A}_{\pi },B\right)\sim N\left({\mu}_{\pi },{\sigma}_{\pi}^2\right)$ [20]. We further standardized the GHD value so it follows a standard normal distribution and evaluated its significance against the normal quantiles.

### Pathway analysis

After we compared the pre−/posttreatment module pairs, we performed pathway-enrichment analysis for module pairs that had topological change between the 2 time points to determine whether the genes from the modules are associated with a certain biological function. Pathway analysis was done using the *gometh* function in *missMethyl* library in R, which conducts hypergeometric tests to determine if the gene sets from the modules contain more genes than expected in a particular pathway [21]. We used *IlluminaHumanMethylation450kanno.ilmn12.hg19* to get annotations and tested 319 pathways in the Kyoto Encyclopedia of Genes and Genomes [22].

## Results

### Module assignment

Six subnetworks, whose module sizes are 210, 177, 123, 112, 91, and 44, were identified in the pretreatment network, while 14,049 probes remained outside of these modules. Each module was assigned a color: pink, blue, brown, yellow, green, and red that are in descending order of module size.

### Module preservation analysis

**Fig. 1:**
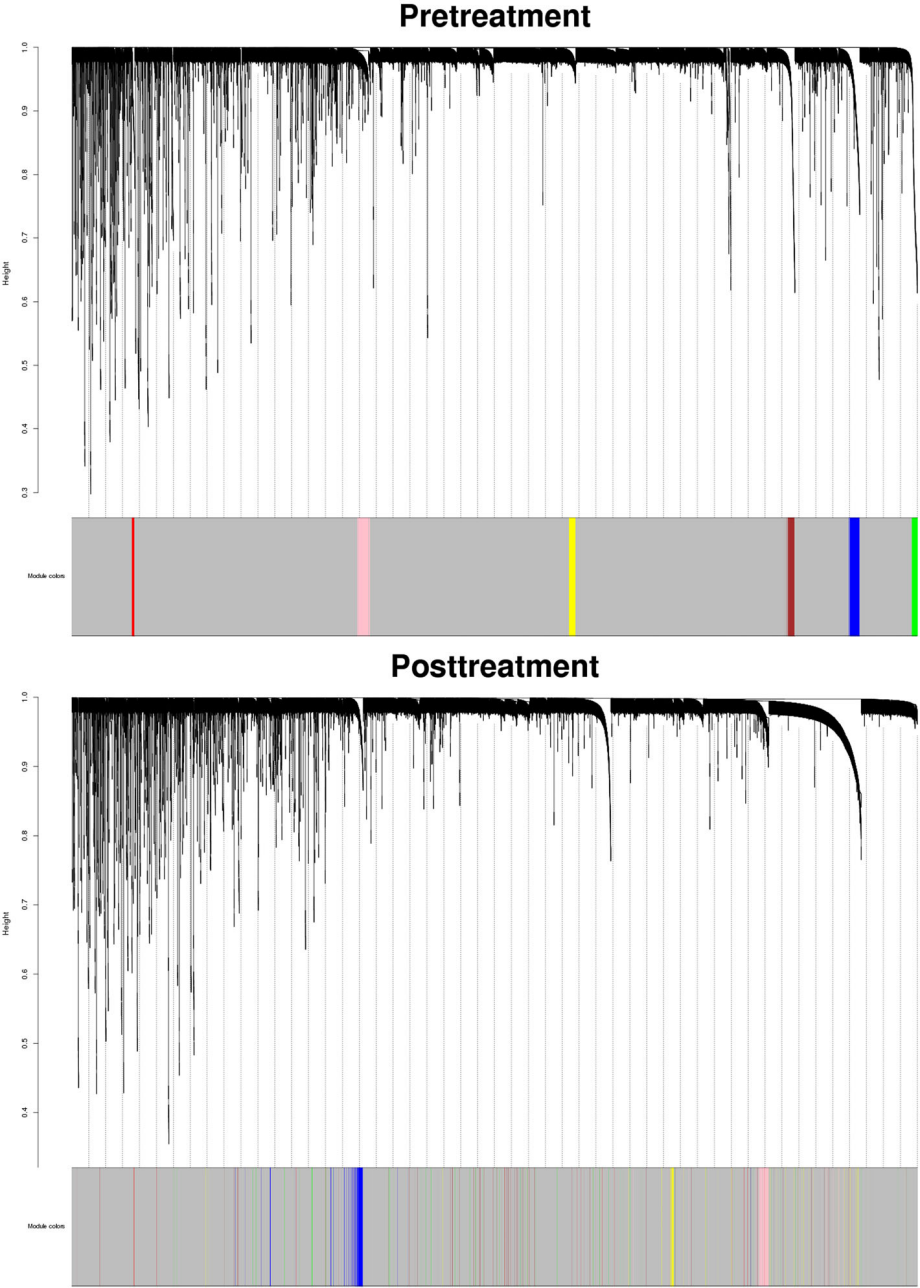
Clustering dendrogram of pre- and posttreatment methylation probe network, together with assigned module colors (pink, blue, yellow, red, brown, and green)

**Fig. 2:**
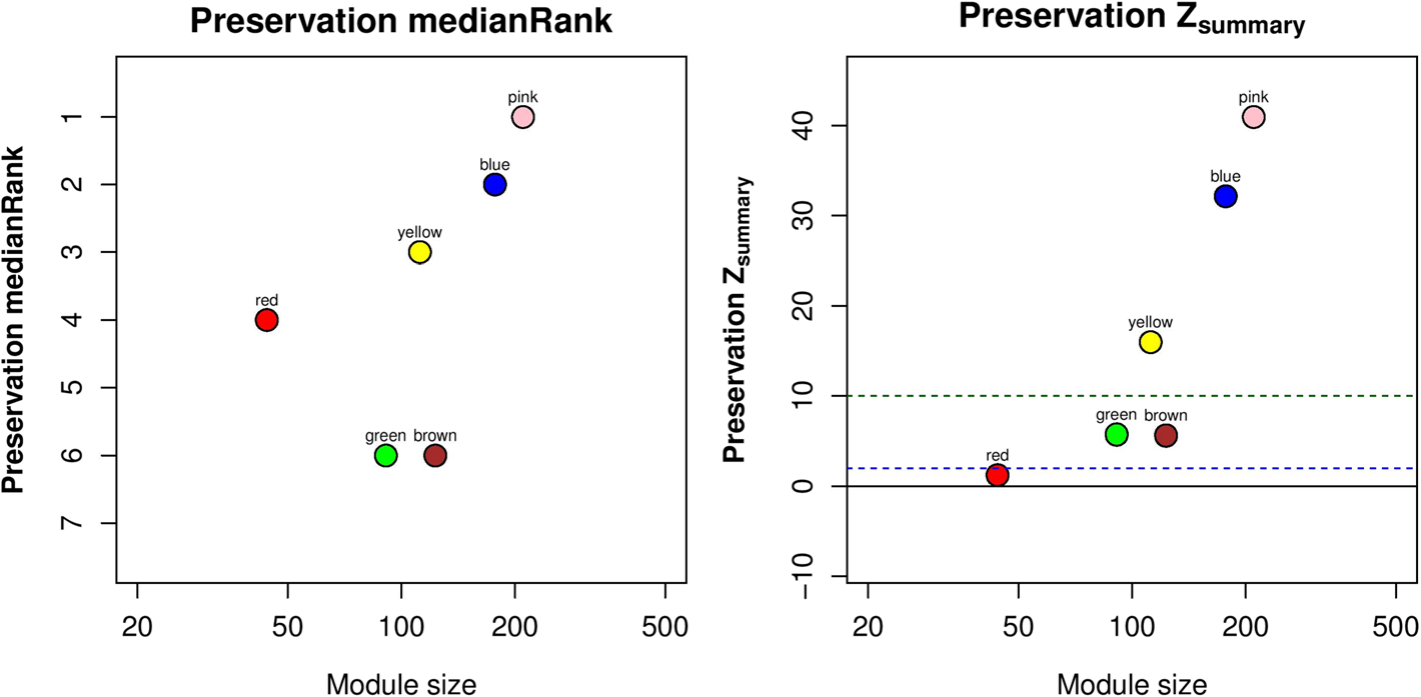
The medianRank and *Z*_*summary*_ statistics of module *preservation* of pretreatment modules in posttreatment modules (*y*-axis) versus module size (*x*-axis)

In Fig. 1, methylation probes assigned to blue, pink, and yellow modules clearly grouped together in the posttreatment network, indicating fairly good preservation for these 3 modules. As seen in Fig. 2, pink, blue, and yellow modules also had a *Z*_*summary*_ > 10 and were ranked at first, second, and third, respectively, when applying *medianRank* statistic, revealing their good preservation in the posttreatment network. Green and brown modules were tied for fifth place, and their *Z*_*summary*_ statistic values were < 10, so it is likely these 2 modules were not preserved between pre- and posttreatment. The red module had a *Z*_*summary*_ of 1.234, which was < 2, and was ranked fourth in terms of *medianRank* statistic.

### GHD

Unlike WGCNA, the GHD method requires a (0,1)-adjacency matrix. We obtained such a matrix by thresholding the absolute value of the correlation matrix obtained from WGCNA. We used a threshold of 0.2, which results in a nearly scale-free network. It is thought that biological networks are scale-free, which are characterized by a small number of highly connected nodes known as hubs that hold the network together [16]. Under the null hypothesis, pretreatment and posttreatment modules are independent, which means that there is topological difference between the two modules. For each pre–post module pair, we calculated the GHD statistic, the mean, and the variance, and computed the corresponding *Z* score and the *p* value.

We used Bonferroni-corrected *p* values to account for multiple testing, ie, a significance threshold of 0.05/6 = 0.00833. Table 1 shows that the pink, blue, and yellow module pairs were not independent, which implies that the pre- and posttreatment modules did not have topological differences. Conversely, the green, brown, and red modules show a differential topological structure between pre- and posttreatment networks. Of note, the red module has a borderline *p* value of 0.009, which is very close to the Bonferroni corrected alpha of 0.00833, suggesting that it is nearly significant in having no topological difference between pre- and posttreatment.

**Table 1:** Results of independence test of each module pair between pre- and posttreatment networks

Modules	GHD	Z score	*p* Value
Pink	0.025	− 11.774	5.307 × 10^−32^
Blue	0.092	−9.384	6.350 × 10^−21^
Yellow	0.059	−8.106	5.225 × 10^−16^
Red	0.016	2.609	0.009
Brown	0.041	0.953	0.340
Green	0.006	0.462	0.643

Null: pre- and posttreatment modules are dependent (there are structural changes); alternative: pre- and posttreatment modules are independent (no structural changes)

We found that rankings of 6 modules from GHD and *medianRank* statistic were similar (ranked first to sixth for module-preservation level: pink, blue, yellow, red, brown, and green) by comparing the results from module-preservation and GHD methods. Although its *Z*_*summary*_ showed the weakest preservation, the red module had a borderline *p* value of showing no topological difference and was ranked fourth; further investigation may be necessary to evaluate module preservation of red module.

### Visualization

As mentioned previously, the red module required further investigation to determine if there is any topological difference between pre- and posttreatment. We examined the structural changes for the red module by making network plots. We used the same threshold of 0.2 as in the first step of the GHD method, and converted each cell of the adjacency matrix from absolute value of the correlation coefficient into a value of 0 or 1. If the dichotomous correlation coefficient between 2 probes was 1, we drew an edge between them.

**Fig. 3:**
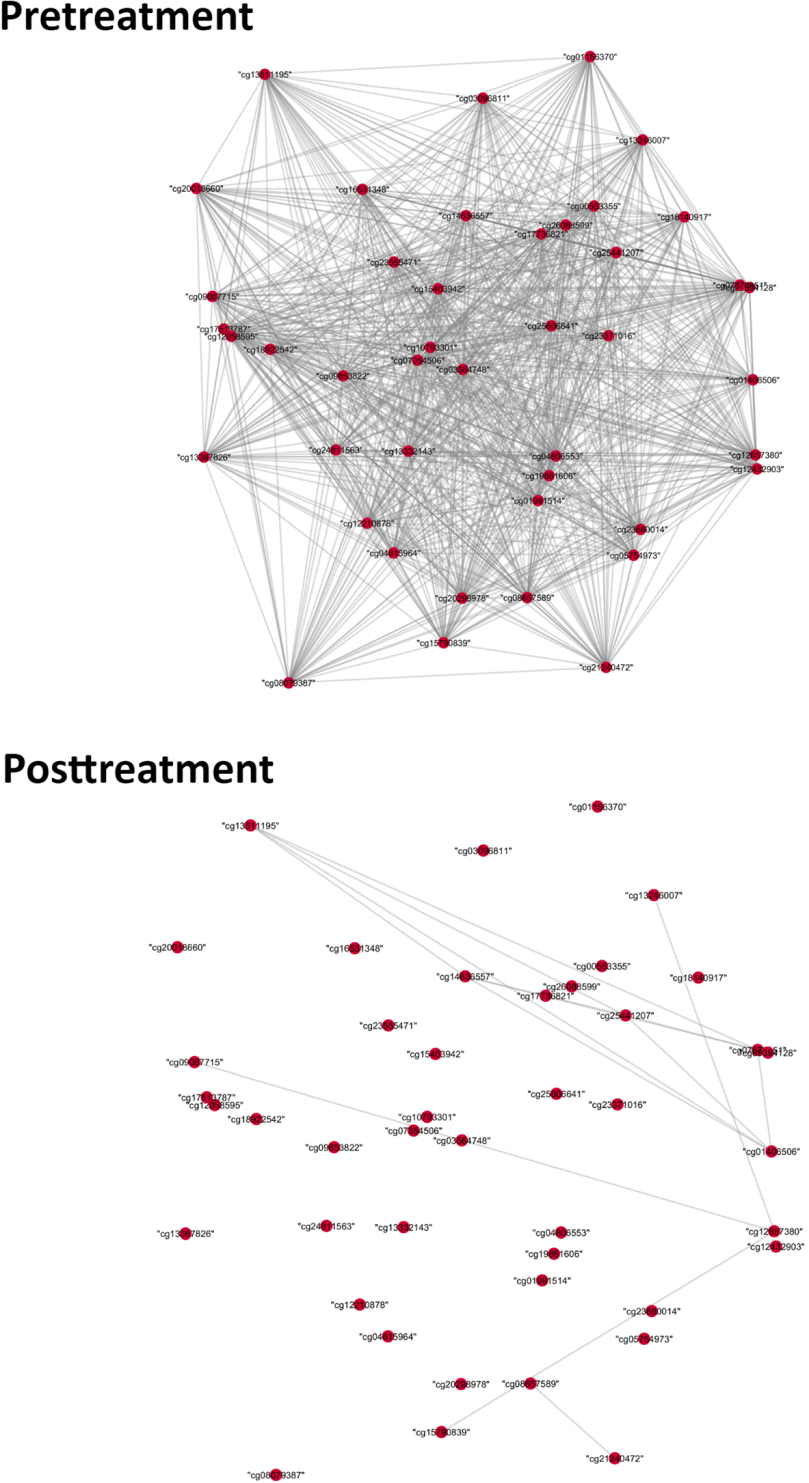
Pretreatment and posttreatment network plots of red module

In the 2 network plots in Fig. 3, there was an apparent difference of network structure between pre- and posttreatment of the red module. The strong relationships between most of the methylation probes in the red module disappeared in the posttreatment network. The network plots of the red module were similar to those of the green and brown modules, indicating that the red module was also differentially methylated between pre- and posttreatment. Further investigation into the probes is needed to determine if the changes were the result of a drug effect. One such potential investigation would be to perform a pathway-enrichment analysis with these modules to determine if methylation probes can be linked to genes related to lipid signaling.

### Pathway analysis

For the 3 differentially methylated modules (green, brown, red), we performed pathway-enrichment analysis to test for overrepresentation. Table 2 shows the results of the pathway analysis for the red, green, and brown modules. Compared to the brown and green modules, the red module is made up of more genes that are involved in lipid signaling and metabolism, which points to overrepresentation in the gene list in the red module. For example, *LPCA1* and *AGPAT3* are known to be involved in encoding enzymes that play a role in phospholipid metabolism [23]. This implies that fenofibrate had a bigger effect on triglyceride levels through methylation probes in the red module, leading to a striking structural change between the 2 time points as is shown in Fig. 3.

**Table 2:** Result of pathway analysis for the red, green, and brown modules

Pathway	# DE	*p* Value
Red module		
Sphingolipid signaling pathway	5	1.81 × 10^−32^
Glycerophospholipid metabolism	4	0.00016
Phospholipase D signaling pathway	4	0.0008
Cyclic adenosine monophosphate signaling pathway	4	0.00149
Choline metabolism in cancer	3	0.00561
Ether lipid metabolism	2	0.01523
Glycerolipid metabolism	2	0.03437
Green module		
Metabolic pathway	6	0.00471
Epstein-Barr virus infection	3	0.1168
Notch signaling pathway	2	0.1168
Pyrimidine metabolism	2	0.1492
Basal cell carcinoma	2	0.1492
Brown module		
Proteoglycans in cancer	6	0.00208
*Salmonella* infection	4	0.00208
Terpenoid backbone biosynthesis	3	0.00208
Pathways in cancer	7	0.00239
Gonadotropin-releasing hormone signaling pathway	4	0.00239

*Pathway* indicates the KEGG pathway being tested; *# DE* is the number of genes that are differentially expressed in our module; and the false discovery rate is the *p* value for overrepresentation

## Discussion

By performing network analyses on triglyceride-associated DNA methylation, we found 2 modules (green and brown) that are differentially methylated between pre- and posttreatment based on the results of both 2 statistics (*Z*_*summary*_ and *medianRank*) in the WGCNA module-preservation method and the GHD method. As the red module has a borderline *p* value of showing no topological difference and was ranked fourth, we created network plots for pre- and posttreatment, separately, and observed that it appeared to be differentially methylated. Therefore, 3 modules (red, green, and brown) were found to have topological difference between pre- and posttreatment.

Comparison between module-preservation and GHD methods should also be considered. Rankings of 6 modules from GHD and *medianRank* statistic were similar (ranked first to sixth for module preservation level: pink, blue, yellow, red, brown, and green). However, the *Z*_*summary*_ statistic showed slightly different results, especially for the red module, meaning the red module has the weakest preservation in terms of *Z*_*summary*_, but it was at the middle level when applying the GHD and *medianRank* statistic. This difference between results of *Z*_*summary*_ and *medianRank* statistic may be a result of *Z*_*summary*_’s sensitivity to module size. Because the red module has a relatively small module size of 44, its lack of preservation based on low *Z*_*summary*_ value may not be as credible as other large modules [19]. Thus, further investigation using network visualization was necessary to evaluate the preservation level of red module.

Some limitations of WGCNA and 2 network comparison methods surfaced during the analysis. Edge direction was not considered in any of the statistics, possibly resulting in loss of information regarding relationships between methylation probes. It would be more telling if we could use a two-step or even more general topological overlap rather than one-step topological overlap to include more information about the neighboring structure around a pair of methylation probes to define the GHD statistic, although it may not be straightforward to prove normality of the sampling distribution under the null.

## Conclusions

We explored the relationship between triglyceride-associated DNA methylation and fenofibrate using WGCNA to construct pre- and posttreatment co-methylation networks and detect topological differences between the 2 networks. Using both module-preservation and GHD methods, we found 3 modules that were differentially methylated in posttreatment. Enrichment analysis of these 3 modules revealed that some of these modules are made up of genes that are involved in phospholipid metabolism, which may provide insight into the effect of fenofibrate on changes in methylation and triglyceride levels. Although we cannot conclude that fenofibrate induced the epigenetic alterations, our network-based approach seems promising for detecting treatment-induced changes in comethylation.
